# Aerobic exercise training in older men and women—Cerebrovascular responses to submaximal exercise: Results from the Brain in Motion study

**DOI:** 10.14814/phy2.15158

**Published:** 2022-02-25

**Authors:** Sonja L. Lake, Veronica Guadagni, Karen D. Kendall, Michaela Chadder, Todd J. Anderson, Richard Leigh, Jean M. Rawling, David B. Hogan, Michael D. Hill, Marc J. Poulin

**Affiliations:** ^1^ Department of Physiology and Pharmacology Cumming School of Medicine University of Calgary Calgary Alberta Canada; ^2^ Hotchkiss Brain Institute Cumming School of Medicine University of Calgary Calgary Alberta Canada; ^3^ Clinical & Translational Exercise Physiology Lab Cumming School of Medicine University of Calgary Calgary Alberta Canada; ^4^ Department of Clinical Neurosciences Cumming School of Medicine University of Calgary Calgary Alberta Canada; ^5^ O’Brien Institute for Public Health Cumming School of Medicine University of Calgary Calgary Alberta Canada; ^6^ Department of Cardiac Sciences Libin Cardiovascular Institute of Alberta Cumming School of Medicine University of Calgary Calgary Alberta Canada; ^7^ Libin Cardiovascular Institute of Alberta Cumming School of Medicine University of Calgary Calgary Alberta Canada; ^8^ Department of Medicine University of Calgary Calgary Alberta Canada; ^9^ Department of Family Medicine University of Calgary Calgary Alberta Canada; ^10^ Department of Community Health Sciences Cumming School of Medicine University of Calgary Calgary Alberta Canada; ^11^ Division of Geriatric Medicine Department of Medicine Cumming School of Medicine University of Calgary Calgary Alberta Canada; ^12^ Faculty of Kinesiology University of Calgary Calgary Alberta Canada; ^13^ Brenda Strafford Foundation Chair in Alzheimer Research Calgary Alberta Canada

**Keywords:** aerobic exercise intervention, aging, cardiorespiratory fitness, cerebral blood flow, cerebrovascular function

## Abstract

Physical inactivity is a leading modifiable risk factor for cardiovascular and cerebrovascular disease, cognitive dysfunction, and global mortality. Regular exercise might mitigate age‐related declines in cardiovascular and cerebrovascular function. In this study, we hypothesize that a 6‐month aerobic exercise intervention will lead to a decrease in cerebrovascular resistance index (CVRi) and to an increase in cerebral blood flow (CBF) and cerebrovascular conductance index (CVCi) during two submaximal exercise workloads (40% VO_2_max and 65 W), intensities that have been shown to be comparable to activities of daily life. Two hundred three low‐active healthy men and women enrolled in the *Brain in Motion* study, completed a 6‐month exercise intervention and underwent submaximal and maximal tests pre‐/post‐intervention. The intervention improved the gas exchange threshold and maximal oxygen consumption (VO_2_max), with no change in heart rate at VO_2_max, during the treadmill VO_2_max test. Heart rate and CVRi decreased from pre‐intervention values during both relative (40% VO_2_max) and absolute (65 W) submaximal exercise tests. Blood flow velocity in the middle cerebral artery and CVCi increased post‐intervention during 40% VO_2_max and 65 W. Changes in mean arterial pressure were found only during the absolute component (65 W). Our study demonstrates that aerobic exercise improves not only cardiorespiratory indices but also cerebrovascular function at submaximal workloads which may help to mitigate age‐related declines in everyday life. Investigation of the mechanisms underlying the decline in cardiovascular and cerebrovascular capacity with aging has important implications for the maintenance of health and continued independence of older adults.

## Graphical abstract

In this study, we looked at how 6 months of aerobic exercise intervention improved cardiorespiratory fitness and cerebrovascular function in healthy older adults. These gains may translate to an improved ability to live independently for older adults.

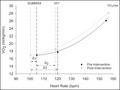

## 1 INTRODUCTION

Declines in cerebral blood flow (CBF) often observed with advancing age are considered to be an important contributor to cognitive decline, as well as cardiovascular and cerebrovascular diseases (Leeuwis et al., 2018). These declines in CBF are observed at rest and in response to various challenges (Tarumi & Zhang, 2018) and they are thought to reflect a decline in cerebrovascular reserve, a term used to describe “the ability of cerebral blood vessels to respond to increased metabolic demand and chemical, mechanical, or neural stimuli” (Davenport et al., 2012). Accordingly, an increase in blood flow to the brain is warranted when demand is increased, with relatively little ensuing change in cerebral blood pressure (Ogoh & Ainslie, 2009; Paulson et al., 1990; Silverman and Petersen, 2021).

Cerebrovascular regulation is a multi‐factorial process influenced by factors such as the arterial partial pressure of CO_2_ (PaCO_2_), cerebral metabolism and neurogenic activity, cardiac output and mean arterial pressure (MAP) (Ainslie & Duffin, 2009; Hoiland et al., 2019; Ogoh & Ainslie, 2009; Phillips et al., 2016). MAP gradually increases with usual aging partially due to arterial stiffening and fibrosis (Fontana, 2018; Franklin et al., 1997). Arterial stiffening leads to a reduced ability of the blood vessels to dilate, which can contribute to a further reduction in brain perfusion (i.e., and oxygen delivery). Cerebrovascular resistance index (CVRi) and cerebrovascular conductance index (CVCi) are traditional indices used to measure the vascular tone and the ability of the cerebrovasculature to react to stimuli (i.e., increases in arterial PCO_2_ or blood pressure; Joyce et al., 2019; Lautt, 1989). Both indices are metrics of vascular tone, but CVRi is normally used when changes in tone are primarily driven by changes in pressure, while CVCi is used when changes in tone are primarily driven by changes in flow (Joyce et al., 2019; Lautt, 1989).

Resting CVRi increases with advancing age as shown in a study that compared young control participants to older ones (Tarumi & Zhang, 2018). Conversely, CVCi has been shown to be decreased in post‐menopausal women when measured at rest and during moderate‐intensity submaximal exercise (Brown et al., 2010).

Regular exercise has been shown to mitigate age‐related declines in cardiovascular and cerebrovascular capacity (Kirk‐Sanchez & McGough, 2014; Ngandu et al., 2015; Pentikäinen et al., 2019; Stensvold et al., 2020). Higher cardiorespiratory fitness benefits both systemic and cerebral circulations, and reduces the adverse neurobiological and cognitive consequences of aging, suggesting that regular exercise may be protective for the brain and may attenuate the age‐related reduction in CBF (Brown et al., 2010; Chapman et al., 2013; Franklin et al., 1997; Gajewski & Falkenstein, 2016; Guadagni et al., 2020; Huggett et al., 2005; Kirk‐Sanchez & McGough, 2014; Tarumi & Zhang, 2018). In a previous study, Murrell et al. (2013) analyzed changes in CBF and cerebrovascular reactivity (i.e., cerebral vasculature response to 5% inspired CO_2_ [PiCO_2_]), during both rest and submaximal exercise (30% and 70% HRR) before and after a 12‐week aerobic exercise intervention in both young and middle‐aged adults. They reported no change in resting middle cerebral artery (MCAv) but an increase in cerebrovascular reactivity (after correcting for a post‐intervention decrease in resting end‐tidal PCO_2_). These results suggest a beneficial role of exercise training on cerebrovascular function in middle‐aged adults.

Previous literature has shown associations between declines in CBF and objective parameters of cardiorespiratory fitness such as maximal oxygen uptake (VO_2_max) (Huggett et al., 2005), and increased risk of neurodegenerative diseases (Ainslie et al., 2008). The reduction in cardiorespiratory function (VO_2_max) and muscular performance associated with advancing age and/or inactivity can contribute to diminished functional capacity (Huggett et al., 2005). In low active or sedentary older adults, functional capacity can drop to levels lower than the critical functional fitness thresholds, which may result in the inability to perform daily life activities and reduce independence (Huggett et al., 2005; Paterson & Warburton, 2010; Taylor, 2014). Indeed, studies investigating the VO_2_ values associated with activities of daily living have identified cut‐off points to predict an individual's ability to perform those activities independently (Huggett et al., 2005; Morey et al., 1998; Paterson et al., 1999). For instance, Paterson and colleagues found that in adults aged 55–86 years, the minimum VO_2_ compatible with independent living was 15.4 and 17.7 ml/kg/min for women and men, respectively (Paterson et al., 1999). Below these cut‐offs, individuals were likely to require assistance (Paterson et al., 1999). The present study extends the concept proposed by Paterson and colleagues, by evaluating cerebrovascular functional capacity at low/moderate exercise intensity below these functional thresholds (Jamnick et al., 2020).

This study is an ancillary sub‐study of the *Brain In Motion study* (BIM), a quasi‐experimental single group pre‐/post‐intervention study (Tyndall et al., 2013). Here, we aim to determine in a large sample of healthy sedentary older adults the extent to which a 6‐month aerobic exercise intervention is associated with improved objective measures of cardiorespiratory and cerebrovascular functions during submaximal exercise at an absolute workload of 65 W (i.e., representing a VO_2_ of approximately 15–17 ml/kg/min; Paterson et al., 1999) and a relative intensity that corresponds to 40% VO_2_max. We hypothesize that a 6‐month aerobic exercise intervention will lead to a decrease in CVRi and to an increase in CBF and CVCi during two submaximal workloads (40% VO_2_max and 65 W). These changes will be above and beyond changes in end‐tidal CO_2_ (PETCO_2_). We propose that such improvements in cardiovascular and cerebrovascular outcomes after the intervention represent increases in functional capacity that have implications for daily life activities.

## 2 MATERIALS AND METHODS

Healthy but underactive participants were recruited through fliers, social media, and world of mouth and provided informed written consent prior to enrolment. The University of Calgary Conjoint Health Research Ethics Board provided ethical approval (CHREB: REB 14‐2284). The data that support the findings of this study are available from the corresponding author upon reasonable request.

### 2.1 Inclusion/exclusion criteria

Participants were required to meet the following criteria to be included in the *Brain In Motion study*: (Tyndall et al., 2018) (1) age between 50 and 80 years at baseline; (2) reporting <30 min of moderate exercise 4 days per week or 20 min of vigorous exercise 2 days per week; (3) a body mass index (BMI) of <35 kg/m^2^; (4) able to walk independently outside as well as up and down at least 20 stairs; (5) not diagnosed with clinically evident cardiovascular or cerebrovascular disease(s), asthma, type I diabetes mellitus and/or another condition that would prevent safe exercise; (6) acquire a score ≥24 on the Montreal Cognitive Assessment (MoCA, Rossetti et al., 2011); (7) non‐smoker for at least 12 months; (8) no major surgery or trauma in the last 6 months; (9) no diagnosis of neurologic disease; and, (10) clearance obtained from their attending health care professional to participate in the study. Prior to being enrolled, participants were assessed by a study physician, and their medications were noted. Participants were excluded from this ancillary study if they did not complete the 6‐month aerobic exercise intervention or had incomplete gas exchange threshold (GET) and VO_2_max data pre‐ and post‐intervention (see “Results” section).

### 2.2 Exercise intervention

Participants took part in a supervised 6‐month aerobic training program that was held three days a week at the Fitness Centre in the Faculty of Kinesiology at the University of Calgary. Each session included a 5‐min warm‐up, aerobic exercise, a 5‐min cool‐down, followed by stretching. As participants progressed through the exercise intervention, the duration of aerobic exercise increased from 20 to 40 min. As well, the exercise intensity increased from 30%–45% up to 60%–70% maximum heart rate reserve (HRR) based on individual VO_2_max results. Polar^®^ heart rate monitors were worn by each participant throughout the session to ensure compliance to their target heart rate zones. Heart rate data were collected and stored for further analysis using the Polar^®^ Team^2^ System. Participants were considered compliant if they attended 85% of the total exercise sessions. If a session was missed, participants were strongly encouraged to complete an unsupervised, “make‐up” session independently, which was recorded using personal workout logbooks. For further explanation on the exercise intervention (see Tyndall et al., 2013 and Hall et al., 2019).

### 2.3 Testing phases

In this report, we focus on data collected immediately prior to the start of the intervention (pre‐intervention) and immediately following the completion of the 6‐month aerobic exercise intervention (post‐intervention). At each phase, participants completed a maximal oxygen uptake (VO_2_max) test, and a cerebrovascular function test during submaximal exercise during separate visits within 1–2 weeks of each other. Several other measurements were collected but they are outside the scope of this report. For further details, please refer to Tyndall et al. (2013).

### 2.4 Cardiorespiratory fitness

Anthropometrics and exercise data were collected in the Clinical and Translational Exercise Physiology Laboratory, Cumming School of Medicine, University of Calgary by Certified Exercise Physiologist (Canadian Society of Exercise Physiology). Anthropometric data were collected prior to completion of the maximal oxygen uptake test and included measurements of participant's height, weight, and skin folds. Following, maximal oxygen uptake (VO_2_max) was determined using a metabolic cart (Parvo Medics TruOne 2400). Ventilatory volumes and expiratory gases were measured during a ramp exercise test on a programmable motorized treadmill (Quinton TM55). Baseline ventilatory measures were obtained during a three‐minute period of quiet standing on the treadmill. Warm‐up measures were obtained during a four‐minute slow walk at a speed of 1.7 mph and a 0% grade. Following the warm‐up period, a combination of small increases in velocity and grade that occurred every 30 s were used to elicit a ramp‐like test according to previously described methods (McInnis & Balady, 1994). Participants were verbally encouraged throughout the test and exercised to volitional fatigue or until the appearance of symptoms indicating the need to terminate the test according to the American College of Sports Medicine's (ACSM) Indications for Terminating a Symptom‐Limited Maximal Exercise Test (Thompson et al., 2010). Recovery measures were obtained for five minutes following the test at a speed of 1.7 mph and grade at 0%. Heart rate was measured with a 12‐lead electrocardiogram system (QStress) which monitored heart rhythm at rest (5 min) prior to, during, and post‐exercise (3 min). Exercising heart rate, blood pressure (manual brachial measurement), and the participants rating of perceived exertion (RPE) value were measured every two minutes during exercise. Peak heart rate and blood pressure values were recorded at maximal effort. VO_2_max was determined from the highest 30‐s average value during the exercise test. Ventilatory thresholds were determined and verified by two independent investigators according to the V‐slope method (Binder et al., 2008). In this report on older sedentary adults, we solely focused on the GET. With incremental exercise intensity, GET is associated with an increase in lactate following which there is a period of isocapnic buffering. At this point VCO_2_ starts to increase out of proportion to the increase in VO_2_ indicating the buffering of lactic acid by bicarbonate, but PaCO_2_ and PETCO_2_ are relatively stable (i.e., there is no respiratory compensation; Beaver et al., 1986; Poole et al., 2021).

### 2.5 Submaximal exercise tests

#### 2.5.1 Relative workload (40% of VO_2_max) and Absolute workload (65 W)

During the submaximal exercise test, participants were seated on a recumbent cycle ergometer (Lode Corival; Lode BV Medical Technology). Participants first underwent ten minutes of resting air breathing to collect baseline resting end‐tidal respiratory values (PETCO_2_ and PETO_2_) with a dedicated software program (Chamber, University Laboratory of Physiology, Oxford, UK) while on a mouthpiece connected to a fine capillary attached to a mass spectrometer (AMIS 200; Innovision). Then a second specialized program (BreatheM v2.40, University Laboratory of Physiology, Oxford, UK) was used to accurately and continuously record PETCO_2_ and PETO_2_ values during the exercise tests with no gas manipulation; participants for the entire duration of the submaximal exercise test simply breathed room air through a mouthpiece with the nose occluded with a nose clip.

Heart rate was continuously measured throughout the submaximal exercise test using a 3‐lead electrocardiogram system (Micromon 7142 B; Kontron Medical).

Beat‐by‐beat blood pressure was measured continuously using a finger pulse photoplethysmography Finometer (Finapres Finometer Pro; Medical Systems) and the finger pressure transducer was positioned at the heart level. A sphygmomanometer (Welch Allyn) was also used to take brachial measurements during rest.

Arterial hemoglobin saturation was measured using finger pulse oximetry (3900p; Datex‐Ohmeda).

Blood flow velocity in the MCAv was non‐invasively measured using a 2‐MHz transcranial Doppler ultrasound (TCD) (Toc Neurovision^TM^; Multigon Industries Inc.; Leeuwis et al., 2018). MCA location was determined by placing the TCD probe above the zygomatic process near the ear, in the temporal region, and using techniques described by Aaslid et al. (1982). During the first testing session, the TCD probe was manually moved and the TCD settings of depth, gain, and amplitude were optimized to find the best signal from the right MCA. Then the probe placement was recorded by tracing the location on the side of the head on a transparent sheet together with the TCD settings used. To ensure accurate placing of the probe and reliability during different sessions the information recorded during the first visit was used post‐intervention.

In the submaximal exercise test, resting values were collected for 5 min before participants started to cycle. In the first exercise stage (6 min) participants cycled at a work rate relative to 40% of their VO_2_max values collected during the maximal oxygen uptake testing previously described. This was followed by a 6‐min rest period. In the second exercise stage, all participants, regardless of sex, cycled at an absolute work rate of 65 W for 6 min. Lastly, participants completed 6 min of rest (i.e., recovery phase).

### 2.6 Data analyses

#### 2.6.1 Cerebrovascular measures

The TCD signals were collected every 10 ms and averaged values were calculated over each cardiac cycle. Data for the last 30‐s interval of each phase of the submaximal exercise test (exercise bouts and rest) were then averaged. MCAv values collected and MAP (obtained by the beat‐by‐beat data from the Finometer) were subsequently used to calculate CVCi and CVRi:

CVRi=[MAP/MCAv]

CVCi=[MCAv/MAP]

Data were then analyzed using IBM SPSS Statistics, version 25.0 (IBM). Pre‐ and post‐intervention descriptive statistics for the sample are reported in Table 1. Paired sample *t*‐tests were used to compare data pre‐/post‐intervention. To examine the contribution of changes in PETCO_2_ to changes in MCAv and CVCi from pre‐ to post‐intervention, we used a series of multiple linear regressions with post‐scores for MCAv and CVCi (in separate models) as dependent variables, pre‐scores as predictors in block one, and changes in PETCO_2_ (ΔPETCO_2_ = post‐intervention−pre‐intervention) as forced confounders. We ran these multiple linear regressions for changes pre‐/post‐intervention at 40% VO_2_max and 65 W, separately. The advantage of these analyses is that they allow for the quantification of how much of the variance is explained by changes in PETCO_2_ by looking at the *r* ^2^ change of the model that considers the covariate.

**TABLE 1:** Pre‐ and post‐intervention descriptive statistics for the sample (*n* = 203)

Variables	Pre‐intervention		Post‐intervention	
	Mean	SD	Mean	SD
Age, years	66.4	6.4	67.0	6.4
BMI, kg/m^2^	26.9	3.7	26.5	3.6
Height, cm	169.3	9.4	169.3	9.4
Weight, kg	77.6	14.4	76.4	14.2
Waist Girth, cm	96.3	11.3	93.1	11.0
MoCA	27.6	1.4		
Education, years	15.9	2.6		
Biological sex	103 F	100 M		

Note

Values are means ± standard deviations (SD); biological sex is expressed as a count.

Abbreviations: BMI, body mass index; MoCA, Montreal Cognitive Assessment.

A value of *p* < 0.05 was adopted as the minimum level of statistical significance, and all analyses were two‐tailed. A Bonferroni correction for multiple comparisons was used with α = 0.05/5 (or 0.01).

## 3 RESULTS

### 3.1 Subject characteristics

Two hundred eighty‐six participants were initially enrolled in the study. Two hundred and thirty‐six participants completed the pre‐intervention tests and 206 completed the 6‐month exercise intervention. A detailed flowchart of the *Brain in Motion* study is published elsewhere (Guadagni et al., 2020; Hall et al., 2019). In this report, we examine the complete data for 203 participants due to missing cardiorespiratory data for three participants (66.4 ± 6.4 years, MoCA 27.6 ± 1.4, mean years of completed education 15.9 ± 2.6, 103 females).

At pre‐intervention, 63 participants reported being on anti‐hypertension medications, 6 participants reported being on anti‐hyperglycemic 11 and 42 participants reports being on lipid‐lowering medications. After the intervention, 65 participants reported being on anti‐hypertension medication, 6 participants reported being on anti‐hyperglycemic and 41 participants reported being on lipid‐lowering medications. Please refer Table 1 for pre‐/post‐intervention descriptive statistics for select characteristics of the participants.

### 3.2 Cardiorespiratory fitness

The GET increased significantly by 4.6% from pre‐ to post‐intervention (17.78 ± 3.39 vs. 18.64 ± 3.36 ml/kg/min, *t*_(202)_ = −5.99, *p* < 0.001). VO_2_max also increased significantly by 7.1% from pre‐to post‐intervention (26.12 ± 5.48 vs. 28.11 ± 5.86 ml/kg/min, *t*_(202)_ = −13.27, *p* < 0.001). No significant differences were found in heart rate at VO_2_max from pre‐ to post‐intervention (see Table 2).

**TABLE 2:** Pre‐ and post‐intervention cardiorespiratory data for all participants

Variables	Pre‐intervention		Post‐intervention		
	Mean	SD	Mean	SD	Significance
GET, ml/kg/min	17.78	3.39	18.64	3.36	<0.001
GET, L/min	1.377	0.359	1.422	0.362	<0.001
HR_GET_, bpm	120.0	17.0	118.5	15.3	<0.001
VO_2_max, ml/kg/min	26.12	5.48	28.11	5.86	<0.001
VO_2_max, L/min	2.029	0.569	2.150	0.598	<0.001
HR_max_, bpm	154.9	14.1	156.7	14.6	0.095
RER	1.19	0.09	1.19	0.08	<0.001

Note

Values are means ± standard deviation (SD).

Abbreviations: GET, gas exchange threshold; HR_GTE_, heart rate at GET; HR_max_, heart rate at maximal oxygen uptake; RER, respiratory exchange ratio; VO_2_max, maximal oxygen uptake.

### 3.3 Responses to submaximal exercise

#### 3.3.1 Relative workload (40% of VO_2_max)

At pre‐intervention participants exercised at an average of 51.0 ± 16.3 W to reach 40% of VO_2_max. Post‐intervention the average workload to reach 40% of VO_2_max increased to 55.2 ± 16.9 W, an 8.2% increase.

**FIGURE 1:**
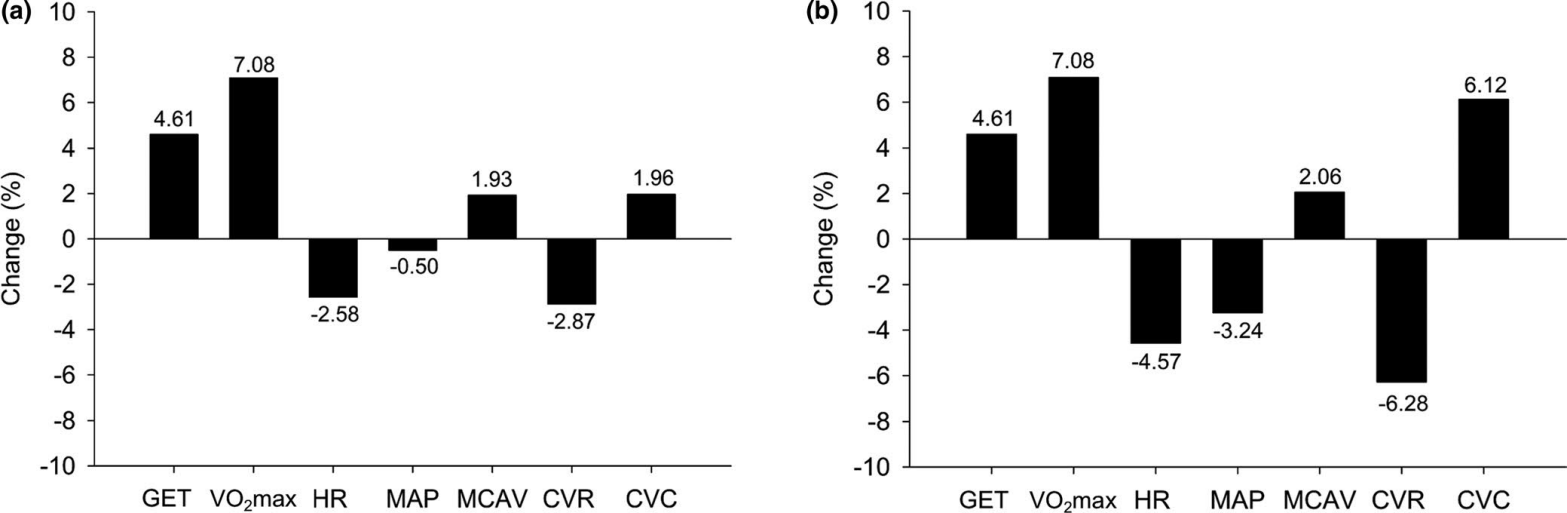
Percent (%) changes from pre‐ to post‐intervention in cardiovascular and cerebrovascular health outcomes during (a) relative (40% VO_2_max) submaximal exercise and (b) absolute (65 W) submaximal exercise

CVRi decreased by 2.9% from pre‐intervention (CVRi; 2.15 ± 0.59 mmHg/cm/s) to post‐intervention (CVRi; 2.09 ± 0.54 mmHg/cm/s), *t*_(199)_ = 2.47, *p* = 0.01), MCAv increased by 1.9% from pre‐ to post‐intervention (55.8 ± 12.4 vs. 56.9 ± 12.1 cm/s, *t*_(199)_ = −2.31, *p* = 0.022) and CVCi increased by 2.0% from pre‐ to post‐intervention (0.50 ± 0.14 vs. 0.51 ± 0.14 cm/s/mmHg; *t*_(199)_ = −2.03, *p* = 0.044). However, the change in CVCi did not remain significant after correction for multiple comparisons (see Tables 3 and 4 and Figure 1).

**TABLE 3:** Pre‐ and post‐intervention cerebrovascular data at rest, relative submaximal exercise (40% of VO_2_max), and absolute submaximal exercise (65 W) for all participants

Variables	Rest		40% VO_2_max		Rest 2		65 W		Recovery	
	Mean	SD	Mean	SD	Mean	SD	Mean	SD	Mean	SD
Pre‐intervention										
HR, bpm	64.7	9.2	93.1^†^	11.8	69.1	10.4	104.9^†,#^	16.4	75.4	11.9
MAP, mmHg	97.9	11.8	113.8^†^	15.2	102.5	12.3	121.4^†,#^	20.0	101.7	13.1
MCAv, cm/s	51.3	11.2	55.7^†^	12.4	49.7	10.8	54.2^†,#^	12.5	48.9	10.6
CVRi, mmHg/cm/s	2.01	0.53	2.15^†^	0.58	2.17	0.60	2.37^†,#^	0.73	2.1	0.61
CVCi, cm/s/mmHg	0.53	0.14	0.50^†^	0.14	0.49	0.13	0.46^†,#^	0.13	0.48	0.13
PETCO_2_, mmHg	34.3	3.2	36.8^†^	3.2	33.4	4.0	35.5^†,#^	4.4	32.7	3.6
PETO_2_, mmHg	90.3	4.3	89.6^†^	3.8	94.6	5.0	92.1^†,#^	5.8	96.5	4.7
Post‐intervention										
HR, bpm	61.5^***^	8.8	90.8^†,***^	10.7	65.6^***^	9.7	100.3^†,#,***^	15.1	71.1^***^	11.2
MAP, mmHg	97.7	11.7	113.2^†^	15.6	102.2	12.6	117.6^†,#,***^	18.8	101.4	14.0
MCAv, cm/s	52.7^**^	11.7	56.8^†,*^	12.0	50.9^*^	11.0	55.4^†,#,*^	11.9	49.8	10.9
CVRi, mmHg/cm/s	1.94^**^	0.50	2.0^†,*^	0.54	2.1^**^	0.52	2.22^†,#,***^	0.62	2.14	0.59
CVCi, cm/s/mmHg	0.54	0.14	0.51^*,†^	0.13	0.50	0.14	0.48^†,#,***^	0.14	0.50^*^	0.14
PETCO_2_, mmHg	33.8^*^	3.0	36.3^†,**^	3.3	33.0	3.5	35.4^#,†^	3.7	32.2	3.6
PETO_2_, mmHg	90.1	4.3	89.4^†^	4.4	94.4	4.7	91.3^†,#,**^	5.4	96.0	5.2

Note

Values are means ± standard deviation (SD); Rest and Rest 2 were significantly different for each outcome, both pre‐ and post‐intervention (all *p *< 0.001).

Abbreviations: bpm, beats per minute; CVCi, cerebrovascular conductance index; CVRi, cerebrovascular resistance index; HR, heart rate; MAP, mean arterial pressure; MCAv, velocity at the middle cerebral artery; mmHg, millimeters of mercury; PETCO_2_, end‐tidal partial pressure of carbon dioxide; PETO_2_, end‐tidal partial pressure of oxygen.

Significant differences found between pre‐ and post‐intervention are represented by asterisk (*p < 0.05, **p < 0.005, ***p < 0.001); ^†^ indicates significant differences from Rest 1 to 40% VO_2_max and Rest 2 to 65 W; ^#^ indicates differences from 40% VO_2_max to 65 W.

**TABLE 4:** Effect sizes and changes (delta pre‐/post‐intervention) in cerebrovascular data at rest, relative submaximal exercise (40% of VO_2_max), and absolute submaximal exercise (65 W) for all participants

Variables	Cohens *D*_z_	Delta
Rest		
HR, bpm	0.34	−3.12
MAP, mmHg	0.02	−0.36
MCAV, cm/s	0.11	1.37
CVRi, mmHg/cm/s	0.14	−0.07
CVCi, cm/s/mmHg	0.13	0.02
PETCO_2_, mmHg	0.13	−0.41
PETO_2_, mmHg	0.06	−0.25
40% of VO_2_max		
HR, bpm	0.20	−2.30
MAP, mmHg	0.03	−0.59
MCAV, cm/s	0.08	1.03
CVRi, mmHg/cm/s	0.10	−0.06
CVCi, cm/s/mmHg	0.07	0.01
PETCO_2_, mmHg	0.12	−0.46
PETO_2_, mmHg	0.04	−0.27
65 W		
HR, bpm	0.29	−4.60
MAP, mmHg	0.19	−3.62
MCAV, cm/s	0.08	1.02
CVRi, mmHg/cm/s	0.19	−0.13
CVCi, cm/s/mmHg	0.14	0.02
PETCO_2_, mmHg	0.02	−0.12
PETO_2_, mmHg	0.14	−0.80

Note

Values are means ± standard deviation (SD).

Abbreviations: bpm, beats per minute; CVCi, cerebrovascular conductance index; CVRi, cerebrovascular resistance index; HR, heart rate; MAP, mean arterial pressure; MCAV, velocity at the middle cerebral artery; mmHg, millimeters of mercury; PETCO_2_, end‐tidal partial pressure of carbon dioxide; PETO_2_, end‐tidal partial pressure of oxygen.

**FIGURE 2:**
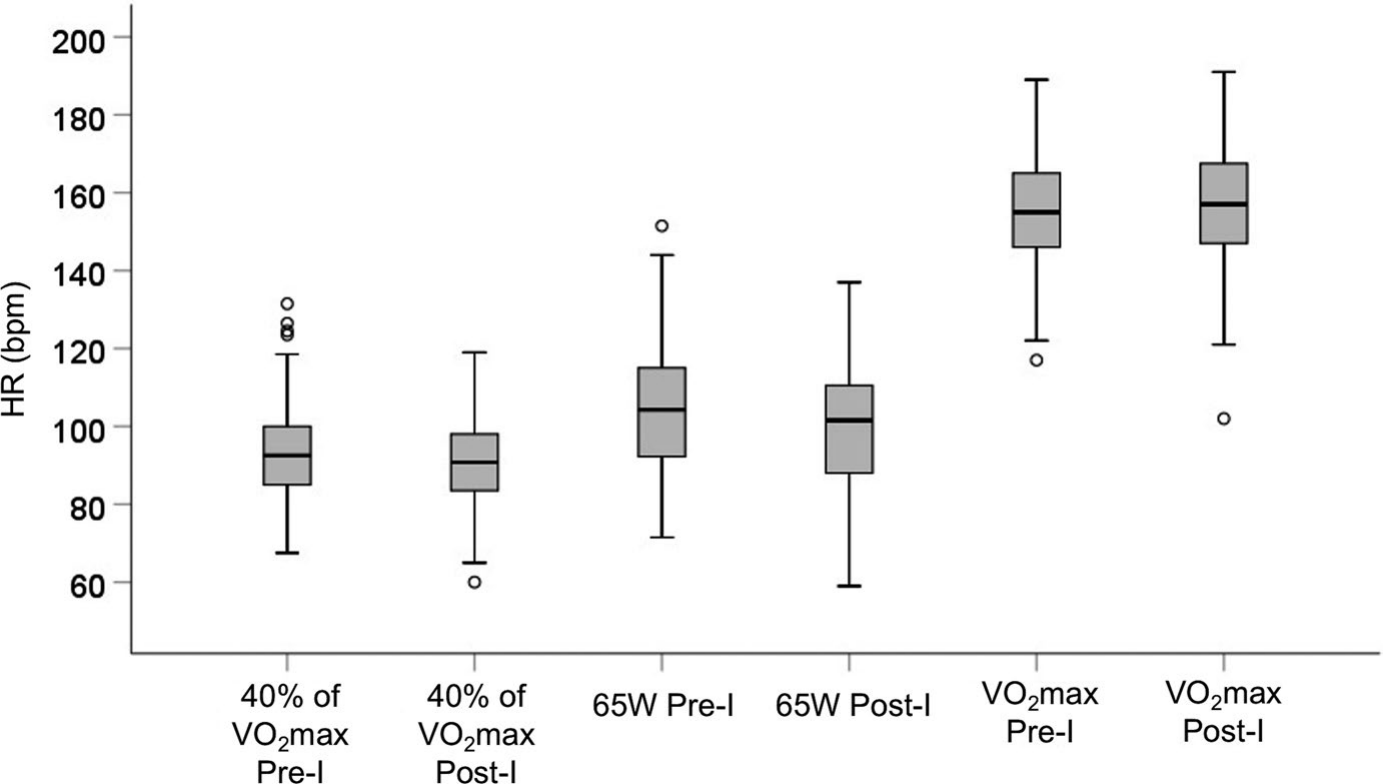
Heart rate (HR) at relative (40% VO_2_max) submaximal, absolute (65 W) submaximal, and maximal (VO_2_max) aerobic exercise pre‐ and post‐intervention (I = intervention)

No significant changes were observed in MAP during relative (40% VO_2_max) submaximal exercise from pre‐ to post‐intervention. HR significantly decreased by 2.6% from pre‐intervention (93.2 ± 11.8 bpm) to post‐intervention (90.9 ± 10.7 bpm), *t*_(202)_ = 3.82, *p* < 0.001 (Figure 2).

#### 3.3.2 Contribution of changes in PETCO_2_ to changes in MCAv and CVCi from pre‐ to post‐intervention at relative workload (40% of VO_2_max)

A Multiple Linear Regression on the change in MCAv from pre‐ to post‐intervention while controlling for changes in PETCO_2_, showed a significant change in MCAv during exercise at a workload of 40% of VO_2_max from pre‐to post‐intervention (*r* = 0.827, *r* ^2^ change = 0.684, *p *≤ 0.001) and a 3.6% contribution of PETCO_2_ to this change (model 2: *r* = 0.848, *r* ^2^ change = 0.036, *p *≤ 0.001).

Similarly, the change in CVCi from pre‐ to post‐intervention while controlling for changes in PETCO_2_, showed a significant change in CVCi during exercise at a workload of 40% of VO_2_max from pre‐to post‐intervention (*r* = 0.763, *r* ^2^ change = 0.582, *p* ≤ 0.001) and a 5.4% contribution of PETCO_2_ to this change (model 2: *r* = 0.797, *r* ^2^ change = 0.054, *p* ≤ 0.001).

#### 3.3.3 Absolute workload (65 W)

CVRi decreased significantly by 6.3% from pre‐intervention to post‐intervention (2.37 ± 0.73 vs. 2.23 ± 0.62 mmHg/cm/s, *t*_(196)_ = 4.29, *p* < 0.001). Unlike relative submaximal exercise, during absolute (65 W) submaximal exercise MAP decreased significantly by 3.2% from pre‐intervention to post‐intervention (121.5 ± 20.1 vs. 117.7 ± 17.9 mmHg, *t*_(198)_ = 3.62, *p* < 0.001). MCAv increased significantly by 2.1% from pre‐ to post‐intervention (54.3 ± 12.5 vs. 55.4 ± 11.9 cm/s, *t*_(196)_ = −2.07, *p* = 0.040). However, the latter change did not remain significant after correction for multiple comparisons. CVCi increased significantly by 6.1% from pre‐ to post‐intervention (0.46 ± 0.14 vs. 0.49 ± 0.14 cm/s/mmHg, *t*_(196)_ = −3.78, *p* < 0.001). Please refer to Tables 3 and 4, and Figure 1. HR significantly decreased by 4.6% from pre‐intervention to post‐intervention (104.9 ± 16.5 vs. 100.2 ± 15.2 bpm), *t*_(199)_ = 6.34, *p* < 0.001 (Figure 2).

#### 3.3.4 Contribution of changes in PETCO_2_ to changes in MCAv and CVCi from pre‐ to post‐intervention at absolute workload (65 W)

A multiple linear regression on the change in MCAv from pre‐ to post‐intervention while controlling for changes in PETCO_2_, showed a significant change in MCAv during exercise at a workload of 65 W from pre‐to post‐intervention (*r* = 0.801, *r* ^2^ change = 0.641, *p* ≤ 0.001) and a 3.4% contribution of PETCO_2_ to this change (model 2: *r* = 0.822, *r* ^2^ change = 0.034, *p* ≤ 0.001).

Similarly, the change in CVCi from pre‐ to post‐intervention while controlling for changes in PETCO_2_, showed a significant change in CVCi during exercise at a workload of 65 W from pre‐ to post‐intervention (*r* = 0.761, *r* ^2^ change = 0.580, *p* ≤ 0.001) and a 2.3% contribution of PETCO_2_ to this change (model 2: *r* = 0.776, *r* ^2^ change = 0.023, *p* ≤ 0.001).

## 4 DISCUSSION

### 4.1 Major findings

This study reports significant improvements in cardiovascular and cerebrovascular indices at the GET and VO_2_max after a 6‐month aerobic exercise intervention in older sedentary adults from the *Brain in Motion study*. Further, we report evidence of increased functional cardiovascular and cerebrovascular capacity at submaximal exercise workloads. The novelty of this study lies in the investigation of the changes in cerebrovascular indices during submaximal exercise at workloads that mimic the demands of activities of daily function. Previous studies have shown favorable cardiorespiratory adaptations and increased time to fatigue in older individuals after aerobic exercise training (Govindasamy et al., 1992; Poulin et al., 1992). Our study provides additional evidence showing favorable effects of aerobic exercise training to the brain in older adults.

We report improvements in cerebrovascular indices during absolute (65 W) submaximal exercise. Specifically, we observed significant decreases post‐intervention of 3.2% in MAP and 6.3% in CVRi, and a 6.1% increase in CVCi. Perhaps not surprisingly, the changes in the cardiovascular and cerebrovascular outcomes during submaximal exercise at the relative workload (40% VO_2_max) were more modest despite an 8.2% increase in workload (51.0 ± 16.3 to 55.2 ± 16.9 W from pre‐ to post‐intervention). These findings provide evidence supporting the importance of aerobic exercise to confer increases in cardiorespiratory fitness, and in turn, improvements in functional capacity as manifested by improved indices of brain health in older adults.

**FIGURE 3:**
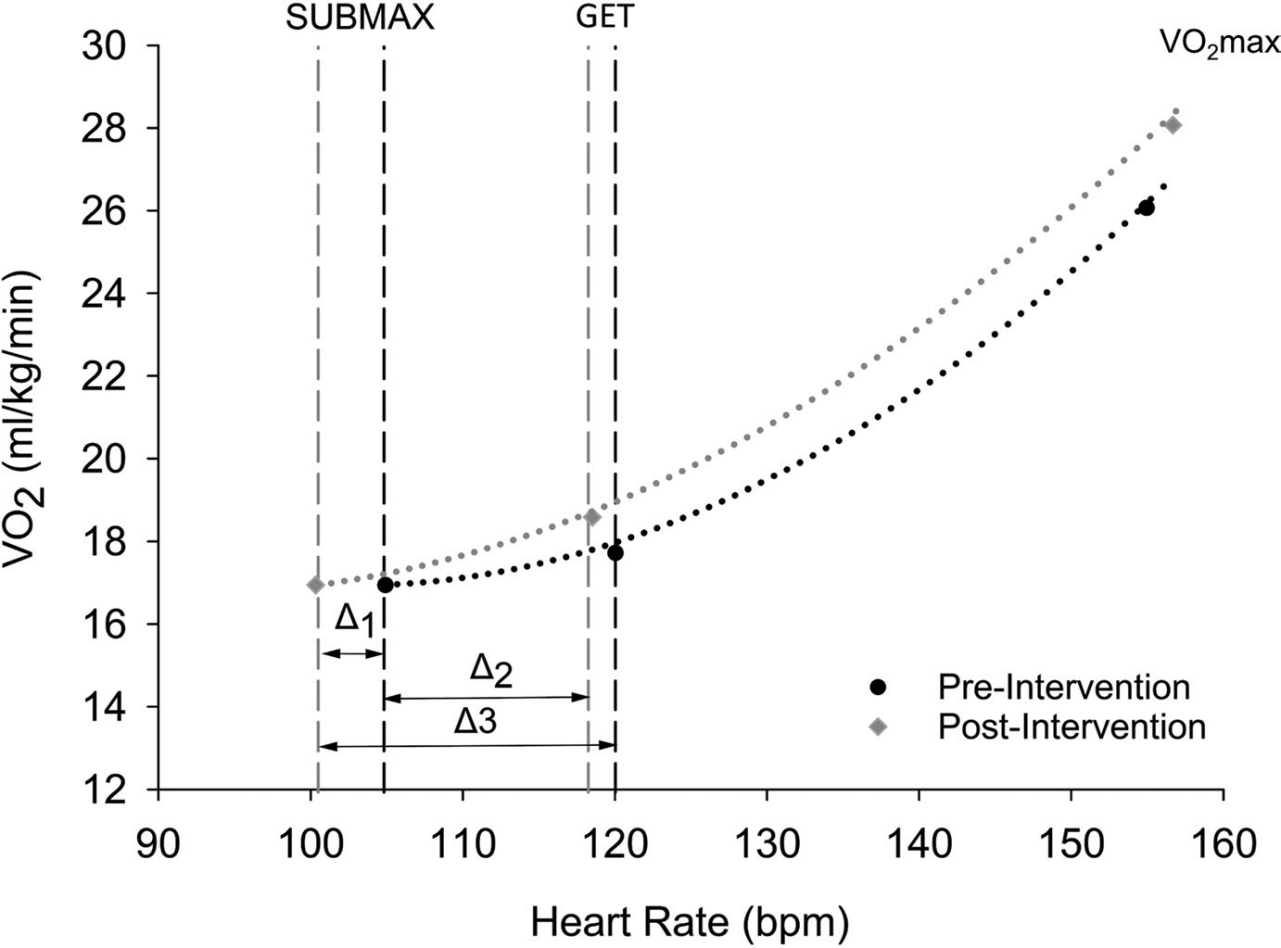
Relationship between heart rate (bpm) and oxygen uptake (VO_2_, ml/kg/min) during submaximal exercise (65 W) before (pre‐intervention, •) and after (post‐intervention, ♦) 6 months of aerobic exercise training. Vertical dash lines represent values before (black) and after (grey) 6 months. The dotted lines follow the VO_2_ values at 65 W, at gas exchange threshold (GET) and VO_2_max before (black) and after (grey) 6 months. Delta 1 (∆_1_) represents changes in submaximal heart rate pre‐ to post‐intervention (HR_∆1_ = 4.6 bpm). Delta 2 (∆_2_) represents changes in HR between 65 W and VT1 pre‐intervention (HR_∆2_ = 15.1 bpm). Delta 3 (∆_3_) represents changes in HR between 65 W and VT1 post‐intervention (HR_∆3_ = 18.2 bpm)

In this study, two different intensities of submaximal exercises were selected to evaluate cerebrovascular functional capacity at exercise intensities that are comparable to activities of daily function. First, selecting exercise intensities below the GET ensured that exercise was performed in the low/moderate‐intensity exercise domain (Jamnick et al., 2020) when there is no respiratory compensation and PaCO_2_ and PETCO_2_ are stable (thus minimizing the possibility of potential confounding factors on our measures of MCAv). Second, 65 W represents a VO_2_ of approximately 15–17 ml/kg/min, which has previously been referred to as a critical threshold for independent living (Paterson et al., 1999). Finally, a relative workload of 40% VO_2_max provided a window through which to evaluate the gains in functional capacity with improved cardiorespiratory fitness post‐intervention. This conceptual framework is depicted in Figure 3, which illustrates the relationship between heart rate (bpm) and oxygen uptake (VO_2_, ml/kg/min) during submaximal exercise (65 W) before (pre‐intervention) and after (post‐intervention) 6 months of aerobic exercise training. Note that the post‐intervention heart rate is lower at 65 W and at the GET, despite a 5% higher VO_2_ at the GET. Moreover, the change in heart rate between submaximal exercise and the GET post‐intervention is greater than at pre‐intervention indicating improved cardiorespiratory fitness (HR ∆_1_ = 15.1 vs. ∆_2_ HR = 18.2 bpm).

A number of studies have described a biphasic association between changes in blood flow to the brain and exercise intensity, characterized by parallel increases in CBF and exercise intensity until ~60% VO_2_max. After this intensity, and when individuals are closer to the GET, the CBF response tends to plateau or even decrease if the exercise intensity increases to the point of hyperventilation induced hypocapnia (Ogoh & Ainslie, 2009; Smith & Ainslie, 2017). In our data, when considering the interplay between some of the main determinants of CBF (i.e., MAP and PETCO_2_) at different exercise intensities, important observations should be made (see Table 3). First, when observing the change in PETCO_2_ from rest to 40% VO_2_ and then 65 W, we notice that PETCO_2_ significantly increases by 7.2% from rest to 40% VO_2_, but then significantly decreases by 3.6% from 40% VO_2_ to 65 W. This effect is maintained post‐intervention, however somewhat attenuated (3.6% decrease in PETCO_2_ from 40% VO_2_ to 65 W pre‐intervention vs. % 2.7 decrease post‐intervention). This suggests a greater cardiorespiratory fitness post‐training when performing exercise at the same intensity (i.e., 65 W). The increase in cardiorespiratory fitness is also confirmed by the blunted increase in HR post‐intervention when exercising at higher intensities (+12.5% at pre‐intervention vs. +10.3%). Concomitantly, we observe that MCAv follows the same biphasic response with an initial increase at 40% VO_2_max (+8%), then a decrease (−2.8%) at a greater exercise intensity (65 W), and the same effect persists after training. Conversely, when observing the changes in MAP with increased exercise intensity, MAP significantly increases by 16.2% from rest to 40% VO_2_max, and by a further 6.6% at 65 W. However, while this effect is maintained post‐intervention it is significantly blunted (6.6% increase in MAP from 40% VO_2_ to 65 W pre‐intervention vs. 3.9% post‐intervention). In turn, CVCi linearly decreases (from rest to 40% VO_2_ to 65 W) in response to increases in MAP, and this effect is reduced after the intervention, a further indicator of improved cardiovascular function after 6 months of aerobic exercise training in older adults.

Previous findings of the effects of aging on cardiovascular outcomes show a 16% decrease in MCAv within three decades of life (40–70 years) which is about 0.53% per year (Vriens et al., 1989). Thus, when comparing previously published normative data with our results, the gains that we observe at post‐intervention appear to represent an improvement of approximately 5 years in brain health—in other words, the MCAv observed post‐intervention represents an average MCAv observed in individuals 5 years younger. The reduction in MAP also showed similar gains. It is known that MAP in older adults is generally higher at both rest and during exercise (Heath et al., 1981). The reduction in MAP observed post‐exercise at 65 W further confirms the beneficial effects of exercise for older adults. Altogether, the exercise‐induced gains attenuate, and may perhaps reverse, age‐related functional declines in CBF and foster healthy brain function (Williams & Leggett, 1989). This may then translate to a greater capacity to perform activities of daily life (Shephard, 2009) such as walking independently, shopping, house cleaning, and other activities that involve a VO_2_ of ~15–17 ml/kg/min (Paterson et al., 1999).

In a recent review, Stillman et al. (2020) describe the effects of exercise on brain health as mediated by multiple mechanisms operating at different system levels. Cellular and molecular effects have been primarily studied in animal models. These studies have shown that exercise increases brain‐derived neurotrophic factor that in turn mediates long term potentiation and neuronal proliferation, vascular endothelial growth factor which supports blood vessels, and insulin‐like growth factor (IGF)‐1, which influences several neural and angiogenic processes (Maass et al., 2016; Voss et al., 2013). From a brain structure perspective (Davenport et al., 2012; Tyndall et al., 2018), exercise has been shown to promote neurogenesis and increase gray matter volume in particular areas of the brain (e.g., the hippocampus) linked to memory and learning (Erickson et al., 2011), increase cortical thickness and volume in the frontal, parietal and temporal cortex (Batouli & Saba, 2017), and increased white matter microstructure (Clark et al., 2019). Improvements in functional connectivity have been also associated with better cognitive function after exercise (Stillman et al., 2016). In this paper, we examine the contribution of exercise training to the ability of the brain to regulate blood flow to ensure adequate delivery of nutrients and oxygen to the different brain areas. Specifically, we found that exercise training increased blood flow velocity and impacted the ability of the brain vasculature to change (increased conductance, decreased resistance) in response to an external stimulus consisting of bouts of submaximal exercise before and after a 6‐month aerobic exercise intervention.

Any age‐related impairments of cardiovascular function, and consequently CBF regulation, may negatively impact the brain's ability to perform cognitive tasks (Barnes & Corkery, 2018; Tarumi & Zhang, 2018). In a recent study from our laboratory (Guadagni et al., 2020), we found improved cerebrovascular function at rest and improved cognition after the 6‐month aerobic exercise intervention. We also found novel associations between changes in the ability of the cerebral vasculature to react to stimuli (i.e., euoxic hypercapnia) and changes in the cognitive domains of executive functions and verbal fluency confirming the role of exercise to maintain brain health. However, the uniqueness of the current study stems from the findings of improvements, after 6 months of aerobic training, in cardiovascular indices at rest in addition to cerebrovascular function during submaximal exercise at workloads that have been shown to be comparable to activities of daily function (Paterson et al., 1999). Previous studies in similar populations have identified the importance of physical activity in mitigating age‐related declines, including sarcopenia and cognitive function (Smith & Ainslie, 2017; Tarumi & Zhang, 2018; Yoo et al., 2018). To our knowledge, this study is the first to provide evidence of improvements in cerebrovascular function during submaximal exercise shown to be comparable to workloads that are required in activities of daily living in a large sample of middle‐aged and older adults after an extensive period of training.

### 4.2 Limitations

The present study has some limitations, which warrant a short discussion. First, the lack of a control group in the study precludes the ability to make firm conclusions on the exclusive role of the aerobic exercise intervention in improving cardiovascular and cerebrovascular indices. However, we have previously published data on lack of changes in cerebrovascular outcomes in the 6 months preceding the intervention (Spencer et al., 2015). These previous findings strengthened the role of the intervention in the improvements observed in this report. Second, this cohort was composed of healthy, well‐educated, mostly Caucasian men and women. As such, our results cannot be generalized to other populations nor to patients with symptom‐limited exercise capacity. Third, the two different submaximal workloads were not randomized, and this may have impacted results and should be addressed in future studies. Fourth, the use of TCD assumes that the cross‐sectional area of the blood vessel being insonated (i.e., the MCA) remains unchanged (Poulin et al., 1996). Moreover, we only measured unilateral (right) MCA while assessment of other large blood vessels and/or global CBF may have yielded different results (Al‐Khazraji et al., 2019). Fifth, the use of finger pulse photoplethysmography to make continuous measures of blood pressure has intrinsic limitations (Maestri et al., 2005), which we have compensated for with the use of a correction factor based on brachial measurements. Sixth, participants were asked to complete an additional unsupervised exercise session once a week and record these and other workouts done on their own in a logbook. These additional sessions were not accounted for in our analyses. Finally, we did not collect any validated direct measure of activities of daily living (ADLs). We however used the Profile of Mood States (POMS) questionnaire to evaluate changes in participants’ mood and vigor from pre‐ to post‐intervention. We found statistically significant decreases (all *p* < 0.05, data not shown) post‐intervention in the subscales of Confusion (pre‐intervention 6.3 ± 2.8, post‐intervention 5.9 ± 2.6), Tension (pre‐intervention 7.9 ± 3.6, post‐intervention 7.3 ± 3.5), and Total Mood Disturbance (pre‐intervention 11.5 ± 20.6, post‐intervention 7.9 ± 20.2) and a significant increase (*p* < 0.001, data not shown) in Vigor (pre‐intervention 18.8 ± 5.4, post‐intervention 19.8 ± 5.1). These changes may be used as surrogate measures for improvements in daily life.

### 4.3 Significance and conclusions

Previous reports on the functional ability of older populations have shown progressive declines in aerobic exercise capacity with age, which is associated with reductions in physical functional capacity, and decreases in independence and quality of life (Christou & Seals, 2008; Huggett et al., 2005; Robinson, 1938). Age‐related declines in cognitive abilities, characterized by chronic and progressive conditions such as dementia, can also reduce the ability to independently perform activities of daily living (Public Health Agency of Canada, 2019). Addressing physical inactivity, an established risk factor of dementia (Livingston et al., 2020), and implementing appropriate exercise interventions, individuals hold promise for reducing the economic and functional burden of dementia.

In conclusion, studies aiming to advance knowledge of the mechanisms underlying the decline in cardiovascular and cerebrovascular capacity with aging have important implications for older adults. Our study suggests that aerobic exercise might improve cardiovascular and cerebrovascular indexes at submaximal exercise levels which are comparable to daily life activities. Future studies are needed to confirm these findings and extend them to other populations and to specific activities of daily living.

## CONFLICT OF INTEREST

All authors report no conflict of interest.

## AUTHOR CONTRIBUTIONS

Sonja L. Lake and Veronica Guadagni share co‐first authorship and equally contributed to the manuscript. Marc J. Poulin, David B. Hogan, Michael D. Hill, and Todd J. Anderson designed the study; Karen D. Kendall and Michaela Chadder conducted the maximal oxygen uptake (VO_2_max) tests and analysed the data for determination of GET and RCP; David B. Hogan, Michael D. Hill, Todd J. Anderson, Richard Leigh, and Jean M. Rawling completed medical assessments and provided medical coverage for the VO_2_max tests; Sonja L. Lake and Veronica Guadagni organized the data and conducted the data analyses. All authors interpreted the data. Sonja L. Lake and Veronica Guadagni drafted the manuscript. All authors read, edited and approved the final draft of the manuscript.
